# Ketamine Therapy in Complex Cases: A Cautionary Tale of Exacerbated Personality Traits and the Crucial Role of Comprehensive Follow-Up and Psychosocial Interventions

**DOI:** 10.1155/2024/2143372

**Published:** 2024-06-17

**Authors:** Jai Ahuja, Luba Leontieva

**Affiliations:** Department of Psychiatry SUNY Upstate Medical University, Syracuse, NY, USA

## Abstract

This case report examines the unexpected increase in suicidal ideation following ketamine infusion therapy in a 75-year-old female with a history of treatment-resistant depression. Despite ketamine's established efficacy in treating depression and acute suicidality, this patient's condition deteriorated posttreatment. The report delves into the patient's complex background, including psychosocial stressors, genetic predisposition to depression, and a history of personality traits that may have influenced her response to ketamine. This case underscores the importance of cautious administration of ketamine, especially in patients with personality disorders, and calls for deeper understanding and individualized treatment plans in mental health care. It is a reminder of the complexities involved in treating mental health conditions and the varying effects of treatments like ketamine on different individuals.

## 1. Introduction

Ketamine has been well-established as a tool in medical practice for decades. Historically, its therapeutic uses have been limited to its functionality as an anesthetic but in recent times, the versatility of ketamine has been explored with an emphasis on its application in the treatment of depression and suicidal ideation. The antidepressant effects of Ketamine were initially explored in a report by Berman et. al [1] and since, multiple studies have explored the therapeutic psychiatric potential of ketamine. There is reasonably strong evidence that supports the antisuicidal effects of ketamine infusion for treatment-resistant depression and suicidality, although these effects have been described as rapid but temporary [2]. Intranasal ketamine has been FDA-approved to treat depression but there are still unresolved issues in this realm, especially with regard to dosing and efficacy [3].

Ketamine's mechanism of action is via antagonism of the is an N-methyl-D-aspartate receptor (NMDAR) and inhibition on GABAergic interneurons leading to a rapid antidepressant effect [4].

The antidepressant effects of ketamine typically manifest about 40 min after infusion, peak around 24 hr later, and begin to diminish, losing effectiveness over placebo after 10–12 days [5]. This temporal profile underscores the need for repeated or semiregular dosing in treating conditions like depression. While intravenous administration is the most common in clinical settings due to its high bioavailability and precise dosing control, alternative methods such as oral, intramuscular, sublingual, and intranasal routes are less resource intensive and offer greater convenience and comfort for patients requiring frequent doses [6]. Additionally, both the psychoactive and therapeutic impacts of ketamine can significantly vary depending on the dose and the method of administration [7].

Research suggests that the antidepressant effects of ketamine are not just due to receptor antagonism but also to the activity of its metabolites, such as (2,6)-hydroxynorketamine (HNK). These metabolites are produced when ketamine is metabolized by the Cytochrome P450 enzymes in the liver [8, 9]. The 2R, 6R stereoisomer of HNK has demonstrated antidepressant-like effects in preclinical models [10, 11].

The relationship between brain-derived neurotrophic factor (BDNF) and tropomyosin receptor kinase B (TrkB), its cognate receptor, is intricately linked to how effectively ketamine works [12]. It is a known fact that BDNF is stimulated by antidepressants [13], and evidence from clinical studies suggests a significant association between the Val allele of Val66Met-BDNF and the heightened antisuicidal and antidepressant responses to ketamine [14]. The sequence of events from when the drug or its metabolite crosses the blood–brain barrier to the upregulation of BDNF expression remains an area of active debate. Additionally, the modest success of other NMDAR antagonists in treating depression underscores the need to reevaluate the importance of this receptor [15].

Depression, in itself, is a multifaceted disorder influenced by a complex interplay of genetic, environmental, and psychosocial factors. Genetic predispositions play a role in an individual's susceptibility to depression and, presumably, their response to treatments like ketamine therapy [16]. For example, variations in genes affecting the metabolic pathways can influence the efficacy and side effects of ketamine, contributing to the varied responses observed among patients. This underscores the importance of considering genetic factors alongside psychosocial stressors when diagnosing and treating depression.

This case explores the paradoxical increase of suicidal ideation after four cycles of ketamine infusion therapy in a 75-year-old female with a history of treatment-resistant depression. Recent literature highlights the growing use of ketamine as a treatment for depression and suicidal ideation and while there is evidence to support the use of ketamine in such clinical scenarios, this case report aims to serve as a point of reference for clinicians who may be considering the unintended consequences of ketamine therapy for their patients.

## 2. Presentation

A 75-year-old female presented to the emergency department (ED) after a suicidal attempt via laceration to her left wrist. The patient had lost five units of blood and required a blood transfusion. The patient has a past psychiatric history of unspecified depressive disorder and a past medical history of breast carcinoma in remission which was treated through chemotherapy. The patient reported that this was her second suicide attempt within 45 days. Between the two suicide attempts, the patient had received four cycles of ketamine infusion therapy which had not resolved their symptoms of depression and suicidal ideation. Her penultimate ketamine treatment was 1 week before her presentation to the ED during which time she had experienced intense emotional flooding, increased suicidal thoughts, and unresolved feelings of worthlessness and despair. She did not have any follow-up from her ketamine treatment provider. Upon admission, a detailed mental status examination was conducted: The patient was an overweight 75-year-old female who appeared younger than her stated age, lying down in bed dressed in a hospital gown, showcasing good hygiene despite left forearm lacerations from the suicide attempt. She maintained appropriate eye contact throughout the evaluation, and her speech was spontaneous with a normal rate, volume, and quantity. Her mood was reported as “I'm Ok” with a dysphoric, tearful yet stable affect that was appropriate and congruent with her stated mood. Thought processes appeared linear, coherent, and goal-directed with the content revealing passive suicidal ideation but no passive homicidal ideation. She displayed no signs of internal preoccupation or hallucinations. The patient was awake, alert, with fair insight and poor judgment.

## 3. Background

Approximately 6 months before the presentation, the patient had moved in with her son from her previous hometown of 30 years. Patient perceived a lack of support from her son's family which resulted in an initial suicidal attempt in October 2023. The patient subsequently underwent four rounds of ketamine infusion therapy with a private psychiatrist for which she paid out of pocket. The treatment did not produce improvement in her mood, she continued to feel depressed and another suicide attempt in December 2023.

The patient first reported feelings of depression after her father's death 15 years prior to the presentation. She did not seek psychiatric intervention at that time and was able to cope through immersion in work. However, a diagnosis of breast carcinoma prompted an early retirement in 2015 from her job as manager for a bank. The lack of a professional life was a source of major distress for the patient who had used her busy professional life as a coping mechanism for multiple worries in her personal life. In April 2020 during the COVID-19-induced lockdown, the patient had her first diagnosed depressive episode prompted by seclusion from her community following which she sought help from a psychiatrist. She was prescribed paroxetine 40 mg and olanzapine 5 mg which was initially effective for her symptoms. However, over the next few years, the patient increasingly started having episodes of depression during which she called her children and expressed suicidal ideation. Her son consequently asked her to relocate interstate to his town of residence to bring her closer to family. However, the patient perceived that she was not getting enough attention from her family. She increasingly voiced feelings of despair and worthlessness along with a lack of hope for the future which led to her two suicide attempts in October and December 2023. Her family reported that the patient was prone to episodes of emotional flooding during which she was increasingly fixated on her feelings of despair and would express low impulse control. He said that she would often tell them that she wants to die and would keep talking about negative instances in her life during this time. She would not be receptive to counseling and would even be rude toward her son and his family when he tried to speak to her during these episodes. As per her family, both of her suicide attempts occurred during these episodes.

## 4. Past Medical History

The patient has a documented history of multiple comorbidities, including diabetes mellitus, hypercholesterolemia, hypertension, hypothyroidism, and osteopenia. Each condition has been effectively managed with ongoing medical oversight, ensuring stability, and control.

Eight years prior to her current presentation, the patient was diagnosed with breast cancer during her tenure as a branch manager. Despite the diagnosis, she continued to work while undergoing chemotherapy. One year after the diagnosis, the cumulative effects of her treatment prompted her to opt for early retirement. Currently, the patient's cancer remains in remission, reflecting the effective management of her condition. This significant medical history, particularly her battle with cancer and its aftermath, has had a considerable impact on her overall health and mental state, especially postretirement when she faced challenges adapting to a life without the structure provided by her career.

## 5. Family History

Family history is significant for depression and suicidal ideation in her granddaughter, suggesting a genetic predisposition to depression. Her father and paternal uncle had alcohol abuse problems.

## 6. Social History

The patient's formative years lacked strong social connections, influencing her emotional development significantly. Her only significant childhood relationship was with her father, whose death left her feeling socially vulnerable. After immigrating from South Asia in the 1990s, she faced numerous challenges, including financial difficulties and a significant personal betrayal when her husband left her for a friend. These experiences contributed to her feelings of isolation and abandonment.

As an adult, her primary social interactions stemmed from her work. Retirement from her career as a bank manager due to breast cancer treatment marked the onset of her depressive symptoms, as she struggled with the loss of her professional identity and daily routine.

Per collateral information, her social life was limited by the exhibition of certain personality traits her entire life including a sense of entitlement and externalization. She had a pattern of not taking accountability for her actions, was judgemental in nature, and frequently commented on others' appearances. She was also prone to using manipulative tactics that were self-serving in nature. Additionally, she would often feel victimized and did not display trust toward other people and had a history of histrionic behaviors in response to minor disagreements. She often exhibited binary thinking, swiftly categorizing situations or people as either entirely good or entirely bad.

### 6.1. Course during Inpatient Treatment

The patient had an intriguing course of admission concerning her depressive symptoms. She was euthymic and her affect and behavior did not match depressive symptoms. She expressed regret for her actions and stated that she would never do anything that could cause her family pain. She was increasingly concerned about her hygiene and appearance. The patient was started on paroxetine 40 mg and olanzapine 5 mg, the latter to aid in sleep and as an antidepressant augment. Given her past positive response to paroxetine, it was chosen as the primary SSRI for treatment. The doses were later increased to 50 and 10 mg, respectively, due to her initial response. This pharmacological treatment was complemented by both individual and group psychotherapy, addressing her personality traits and their impact on her coping mechanisms for dealing with life and loneliness. The therapeutic combination led to significant improvements, making alternative treatments like electroconvulsive therapy (ECT) or lithium unnecessary. Additionally, since she was stabilizing on paroxetine, switching to another SSRI like fluoxetine was not deemed necessary.

During her stay, the patient exhibited manipulative behavior; she attempted to trade coffee for compliance in allowing the medical staff to obtain a thorough history and follow-up. This action reflects her longstanding pattern of manipulating situations to her perceived benefit, often at the expense of appropriate boundaries. Her transference toward specific providers also became evident when she expressed that she would only comply with medical recommendations if a particular provider shared personal contact details with her. When her request was denied, she turned vindictive, showcasing her inability to handle rejection and her propensity to react negatively when boundaries were enforced. These incidents are consistent with the histrionic and dependent traits diagnosed upon discharge.

She also showed a strong attachment to certain caregivers, often blurring professional boundaries in her interactions. This behavior included requests for personal favors and social interactions beyond the scope of therapeutic engagement, which were gently but firmly declined by the staff.

The patient's family provided additional insights into her personality, noting her longstanding tendencies to manipulate, victimize herself, and lack accountability for her actions. They described her as someone who “holds grudges,” “cares deeply about appearances,” and is “distrustful of others,” often feeling wronged by people. He emphasized that she has historically lacked the ability to introspect, which has been a barrier to forming meaningful relationships and has contributed to her interpersonal conflicts.

The patient was discharged on the sixth day after admission along with follow-ups planned with an outpatient psychiatrist and therapist plus social support resources. At discharge, the mental status examination showed she was cooperative, using a walker, and dressed appropriately. She was alert, oriented, and her cognitive functions were intact. Her mood was stable and her affect was euthymic with slight anxiety, but appropriate to the situation. Discharge diagnoses included Unspecified Depressive Disorder, Unspecified Anxiety Disorder, and Personality Disorder with dependent and histrionic traits. These reflect her persistent interpersonal challenges and manipulative behaviors observed during her stay. By the sixth day, the patient felt better and was ready to leave the hospital, as she was no longer suicidal. This aligns with acute psychiatric unit discharge criteria in the USA, which stipulate that a patient may be discharged if they are no longer a danger to themselves or others and have outpatient referrals in place.

## 7. Discussion

This is a complicated case of treatment-resistant depression and suicide attempts in a 75-year-old female who recently received ketamine treatment. Patient also had personality characteristics that became exacerbated as she aged and significantly contributed to diminished ego strength and resiliency with consequent despair.

Given the complex interplay between patient's psychosocial, genetic, and biological factors, there are multiple theories including the following points.

### 7.1. Psychosocial Factors and Diminished Ego Strength

The patient's unique psychosocial background and diminished ego strength play crucial roles in her response to ketamine therapy. Growing up with limited social connections and primarily relying on a close bond with her father set a pattern of dependency and emotional vulnerability. This early attachment dynamic likely contributed to her struggles with relationship stability in adulthood, a factor critical in understanding her varied responses to treatment. Psychologically, her fragmented sense of self, poor impulse control, mood lability, and narcissistic traits, as per Kernberg's level of personality organization [17], reflect a borderline level of personality organization.

The patient's life took a significant turn with her move to the United States, where she built a successful career as a banker. However, this trajectory was disrupted by her early retirement due to breast cancer, divorce, and the subsequent loss of friends and acquaintances. Aligned with Erikson's theory of psychosocial development, these significant life events likely precipitated a sense of despair as the patient encountered the integrity vs. despair stage, characteristic of late adulthood [18]. Previously, the patient demonstrated considerable ego strength, evidenced by her ability to raise two children on her own, successfully navigate a career in banking from a part-time employee to a manager, and cope with significant life challenges. However, her ego strength has waned over time due to impactful life events such as a cancer diagnosis and a divorce. These transitions have markedly diminished her once robust resilience, increasing her vulnerability to depressive episodes. According to Erikson's theory, such profound changes in late adulthood can often trigger a crisis of integrity vs. despair, profoundly affecting her psychological stability and her response to therapeutic interventions like ketamine. The accumulation of these life stressors—her early retirement, failed marriage, social isolation, and health issues—appear to have precipitated a profound state of despair.

Her relocation interstate in 2023 further exacerbated her vulnerability to depression. The loss of community and a supportive environment disrupted her social structure, eventually leading to a significant depressive episode.

The patient's emotional flooding, characterized by intense and prolonged negative emotions, is particularly significant. These episodes contribute to her rudeness toward family members, difficulty maintaining stable relationships and eventually her suicide attempts. Emotional flooding is a manifestation of her struggle with vulnerability and fear of betrayal, highlighting the profound impact of psychosocial factors on her depressive episodes.

Regarding the consistency of monitoring by the psychiatric team, it is important to clarify that while the patient has been under continual care, variations in follow-up intensity and personnel may have influenced the continuity of observations. To ensure a comprehensive understanding of treatment response, efforts have been made to maintain consistent oversight, though challenges remain in standardizing this across different care settings.

### 7.2. Genetic Factors

Genetics plays a significant role in predisposing individuals to depression. In this case, the patient's genetic makeup is marked by vulnerabilities on two fronts. First, there is a family history of depression, evident in the patient's granddaughter's struggles with suicidal ideation. This suggests a genetic predisposition within the family, increasing the patient's susceptibility. Second, the family history also reveals a tendency toward addiction, with her biological father and brother having a history of alcohol use. The co-occurrence of depression and addiction within families underscores the intricate relationship between these conditions. Shared genetic factors contribute to the likelihood of both conditions emerging, potentially affecting the patient's mental health.

While the patient herself has not exhibited substance abuse tendencies, her genetic predisposition could have influenced her vulnerability to depression [16]. This genetic backdrop sets the stage for understanding her depressive episodes but is only one piece of the puzzle.

### 7.3. Treatment-Related Factors

In the context of her treatment, the patient's experience with ketamine therapy raises intriguing questions. While ketamine is recognized for its effectiveness in managing acute suicidality, the patient's second suicide attempt occurred shortly after completing this treatment.

In recent times, ketamine has been well established as a treatment for depression and acute suicidality. Subanaesthetic ketamine is the sole antidepressant approved by the FDA that exhibits rapid efficacy within hours, rather than weeks, hence holding the potential to revolutionize the treatment of individuals at risk of suicide [19]. The reductions in depression and anxiety that follow ketamine infusion are associated with, but may not entirely account for, the decreases in suicide ideation, and further research is necessary to examine the precise impact of ketamine on suicidal ideation [20].

There is evidence indicating that anxiety experienced during ketamine infusions is linked to unfavorable treatment outcomes in individuals with major depressive disorder. This emphasizes the significance of taking into account patients' subjective experiences and suggests the potential for a predictor of how individuals may respond to therapy based on their unique characteristics [21].

Previous studies indicate that the rates of symptomatic deterioration linked to intravenous ketamine treatment for treatment-resistant depression (TRD) are minimal and comparable to traditional antidepressants. Research conducted at the Canadian Rapid Treatment Centre of Excellence found that the frequency of clinically significant worsening ranged from 1.83% to 5.49%, with no discernible pattern over time [22].

Although there have been reports of a quick reduction in thoughts of suicide [23] and a decrease in suicidal thinking following the administration of ketamine [24], it is possible that the increased suicidal thoughts experienced by certain vulnerable individuals were really caused by anxiety and increased agitation after the treatment. The interaction between anxiety and ketamine-induced dysphoria could have exacerbated her distress, leading to suicidal thoughts. This is supported by studies that link comorbid anxiety to increased suicide risk.

Another hypothesis regarding our patient's worsening symptoms postketamine infusion could be related to the interaction of comorbid psychiatric disorders and the acute effects of ketamine. Individuals with personality disorders might experience prolonged or intensified dysphoria after ketamine administration. While our patient did not have a diagnosed personality disorder she exhibited narcissitic, histrioninc, and borderline traits. These vulnerabilities could have made her susceptible to postinfusion anxiety, potentially contributing to her suicidal intent [25].

Ketamine's impact on mnemonic processes and conditioning may disturb the recall, rumination, and reconsolidation of traumatic memories, which could be relevant to our patient's case. The sensory information that patients are experiencing while receiving ketamine may undergo enhancement, reduction, or alteration [26]. If ketamine disrupted her memory processes, it might have triggered distressing recollections or intensified her preexisting emotional distress, potentially contributing to her suicidal intent.

Additionally, patients with personality disorders may respond differently to ketamine treatments, as demonstrated by another report of a patient with MDD and Borderline Personality Disorder who experienced an increase in impulsive and suicidal behavior after esketamine treatment [27]. The brief report highlighted how ketamine treatment may potentially lead to unintentional symptoms such as impulsive behavior and emotional dysregulation.

While there are other reports of ketamine being beneficial for personality disorders, especially borderline personality disorder [28], contrasting reports such as this case have also been published in the literature which necessitates a thorough evaluation of the risks and benefits of pharmacological treatment such as ketamine. Other mechanisms to navigate emotional regulation such as dialectical behavioral therapy [29] should also be explored in such complex patients. If treatments like ketamine infusion therapy are administered, a comprehensive follow-up regimen is encouraged to be established to prevent undesired consequences of therapy.

An inherent limitation of this study is the unavailability of specific psychiatric evaluations conducted during the ketamine treatment process. The patient received therapy out-of-state with a different provider, thus precluding access to formal psychiatric assessments. Consequently, the data presented in the case presentation section primarily rely on subjective reports from the patient, and family members, which may introduce bias and limit the reliability of the findings.

## 8. Conclusion

In summary, this report on a 75-year-old woman dealing with repeated episodes of unspecified depressive disorder sheds light on the intricate and diverse aspects of treating depression and suicidal tendencies, with a focus on ketamine therapy. The unexpected rise in suicidal ideation postketamine treatment illustrates the complexity of mental health care and the varying effects of treatments on different people. This case underlines the need for a thorough approach in treating resistant depression, taking into account genetic factors, personality characteristics, environmental influences, social situation, and personal reactions to medications. It also highlights the critical need for careful, individualized treatment plans, particularly with newer treatments like ketamine. The patient's distinct mix of personal history, personality characteristics, genetic factors, and mental profile highlights the delicate balance of various elements in mental health care. Ketamine providers need to stay connected with patients' primary psychiatric team and take into account postketamine treatment follow-up to avoid potential problems. We urge providers to proceed with caution and take personality disorders and traits into account when choosing to administer this treatment. Ongoing research and clinical practice should strive to deepen understanding of these complexities to develop more effective treatment plans for individuals facing similar challenges, ensuring both their safety and the effectiveness of their treatment.

## Data Availability

The underlying data supporting this case report are from previously reported studies and datasets, which have been cited.
